# Correction: Otopetrin-1: A sour-tasting proton channel

**DOI:** 10.1085/jgp.20181200305102018c

**Published:** 2018-06-04

**Authors:** I. Scott Ramsey, John A. DeSimone

Vol. 150, No. 3, March 5, 2018. 10.1085/jgp.201812003

After publication of this article, several errors were brought to our attention, which we have corrected as follows, with bold indicating insertions and strikethrough indicating deletions.

Both the HTML and PDF versions of the article have been corrected. These errors appear only in print and PDF versions downloaded on or before May 15, 2018.

“Using expression profiling in murine sour taste cells to identify Otopetrin-1 (Otop1) as a proton channel, Tu et al. (2018) show that heterologous overexpression of related proteins from human, mouse, and *Drosophila melanogaster* is sufficient to confer an ensemble of novel H^+^-permeable conductances with varying H^+^ selectivity and pH dependencies.”

“For example, pH_i_ decreases rapidly when pH_o_ is lowered, and the potency for current block by Zn^2+^ (IC_50_ = 200 µM in mOtop1 at pH_o_ 5.5) is similar to the native H^+^ current in sour taste cells (Chang et al., 2010; **Bushman et al., 2015**Horio et al., 2011).

“Tu et al. (2018) further showed that Otop1 is expressed in PKD2L1-YFP^+^ (but not TRPM5-GFP^+^) mouse circumvallate taste cells **and that proton currents in taste cells from Otop1 mutant mice are significantly smaller than those from WT**. Together, the new data present a compelling argument that Otop1 forms an entirely new kind of proton-selective ion channel protein.”

“In rats and mice, chorda tympani (CT) neural responses to HCl are modulated by voltage applied across the lingual surface and blocked by divalent cations including Zn^2+^, whereas acetic acid responses are affected by neither voltage nor Zn^2+^ (DeSimone et al., 2012, 2015; Bushman et al., 2015).”

“Micromolar [Zn^2+^] is well established as a blocker of the H^+^ conductance in sour taste cells, and the proton entry mechanism for strong acids is therefore hypothesized to require a proton channel protein (Chang et al., 2010; DeSimone et al., **2011**2012, 2015).”

“Otop1 represents the best candidate yet for the **sour taste receptor** Zn^2+^-sensitive H^+^ channel in sour taste cells, and the development of genetically modified animals lacking Otop1 will undoubtedly enable a direct test of this hypothesis in the near future.”

“Intracellular acidification resulting from weak acid stimuli triggers an increase in intracellular-free Ca^2+^ ([Ca^2+^]_i_) via release from intracellular stores and/or influx through channels in the basolateral membrane that drives neurotransmitter release (**Richter et al., 2003;** Fig. 1).”

Richter, T.A., A. Caicedo, and S.D. Roper. 2003. Sour taste stimuli evoke Ca^2+^ and pH responses in mouse taste cells. *J. Physiol.* 547:475-83. https://doi.org/10.1113/jphysiol.2002.033811

“Naturally occurring Otop1 mutations (*tlt* and *mlh*) cause vestibular defects, presumably by altering Otop1 trafficking **(Hurle et al., 2003; Kim et al., 2011)**.”

Hurle, B., E. Ignatova, S.M. Massironi, T. Mashimo, X. Rios, I. Thalmann, R. Thalmann, and D.M. Ornitz. 2003. Non-syndromic vestibular disorder with otoconial agenesis in tilted/mergulhador mice caused by mutations in otopetrin 1. *Hum. Mol. Genet.* 12:777–789. https://doi.org/10.1093/hmg/ddg087

Kim, E., K.L. Hyrc, J. Speck, F.T. Salles, Y.W. Lundberg, M.P. Goldberg, B. Kachar, M.E. Warchol, D.M. Ornitz. 2011. Missense mutations in Otopetrin 1 affect subcellular localization and inhibition of purinergic signaling in vestibular supporting cells. *Mol. Cell. Neurosci.* 46:655–661. https://doi.org/10.1016/j.mcn.2011.01.005

“However, more studies are needed to rule out other possible **explanations**mutations, and behavioral tests involving sour taste in *tlt* and *mlh* mice remain to be performed.”

“For example, the ability of Zn^2+^ to modulate voltage-dependent gating in the Hv1 proton channel requires two extracellularly accessible His residues (Ramsey et al., 2006), and the pH_o_ sensitivity of Zn^2+^ block **of H**^**+**^ **currents in taste cells (Bushman et al., 2015)**in Otop1 suggests that His side chains could also be involved in Zn^2+^ coordination.”

